# Arginase 1 Mediates Increased Blood Pressure and Contributes to Vascular Endothelial Dysfunction in Deoxycorticosterone Acetate-Salt Hypertension

**DOI:** 10.3389/fimmu.2013.00219

**Published:** 2013-07-29

**Authors:** Haroldo A. Toque, Kenia P. Nunes, Modesto Rojas, Anil Bhatta, Lin Yao, Zhimin Xu, Maritza J. Romero, R. Clinton Webb, Ruth B. Caldwell, R. William Caldwell

**Affiliations:** ^1^Department of Pharmacology and Toxicology, Georgia Regents University, Augusta, GA, USA; ^2^Department of Physiology, Georgia Regents University, Augusta, GA, USA; ^3^Vascular Biology Center, Georgia Regents University, Augusta, GA, USA

**Keywords:** arginase, endothelial dysfunction, DOCA-salt, fibrosis, hypertension

## Abstract

Enhanced arginase (ARG) activity has been identified as a factor that reduces nitric oxide production and impairs endothelial function in vascular pathologies. Using a gene deletion model, we investigated involvement of arginase isoforms arginase 1 and 2 (ARG1 and ARG2) in hypertension and endothelial dysfunction in a mineralocorticoid-salt mouse model. Hypertension was induced in wild type (WT), partial ARG1^+/−^ knockout (KO), and complete ARG2^−/−^ KO mice by uninephrectomy and deoxycorticosterone acetate (DOCA)-salt treatment for 6-weeks. (Control uninephrectomized mice drank tap water.) After 2 weeks of DOCA-salt treatment, systolic blood pressure (SBP) was increased by ∼15 mmHg in all mouse genotypes. SBP continued to rise in DOCA-salt WT and ARG2^−/−^ mice to ∼130 mmHg at 5–6 weeks, whereas in ARG1^+/−^ mice SBP waned toward control levels by 6 weeks (109 ± 4 vs. 101 ± 3 mmHg, respectively). DOCA-salt treatment in WT mice increased vascular ARG activity (aorta by 1.5-fold; mesenteric artery (MA) by 2.6-fold and protein levels of ARG1 (aorta: 1.49-fold and MA: 1.73-fold) vs. WT Sham tissues. ARG2 protein increased in WT-DOCA MA (by 2.15-fold) but not in aorta compared to those of WT Sham tissues. Maximum endothelium-dependent vasorelaxation to acetylcholine was significantly reduced in DOCA-salt WT mice and largely or partially maintained in DOCA ARG1^+/−^ and ARG2^−/−^ mice vs. their Sham controls. DOCA-salt augmented contractile responses to phenylephrine in aorta of all mouse genotypes. Additionally, treatment of aorta or MA from WT-DOCA mice with arginase inhibitor (100 μM) improved endothelium-mediated vasorelaxation. DOCA-salt-induced coronary perivascular fibrosis (increased by 2.1-fold) in WT was prevented in ARG1^+/−^ and reduced in ARG2^−/−^ mice. In summary, ARG is involved in murine DOCA-salt-induced impairment of vascular function and hypertension and may represent a novel target for antihypertensive therapy.

## Introduction

Arterial hypertension remains a major risk factor for cardiovascular disease morbidity. It affects about 26% of the adult population (1). Despite current drugs being effective in many patients, a large number of uncontrolled patients are still very evident today. Hypertension is associated with physiological and biochemical changes in the vessel wall, characterized by turbulent blood flow, fluid shear stress, vascular remodeling, and endothelial dysfunction. During the past few years, many studies have demonstrated that nitric oxide (NO) pathway is a major regulator of cardiovascular functions, and evidence has accumulated that enhanced arginase (ARG) activity is involved in the pathogenesis of several cardiovascular disorders, including hypertension (2, 3).

Arginase is a crucial manganese metalloenzyme in the hepatic urea cycle that catalyzes conversion of l-arginine to ornithine and urea. It exists in two isoforms, arginase 1 (ARG1) and arginase 2 (ARG2). Each is encoded by a separate gene and found in vascular tissues, endothelial, and smooth muscle cells, but their distribution is vessel- and species-dependent (4–6). In the endothelium ARG activity appears as a critical regulator for NO production by competing with endothelial NO synthase (eNOS) for l-arginine (7). Related studies have shown that increased ARG activity/expression is involved in many vascular pathologies including atherosclerosis (6, 8), aging (9), diabetes (10, 11), and hypertension (12–14). Blood pressure is mainly regulated by the tone of resistance vessels. Increased ARG activity and diminished NO bioavailability are observed in conduit and resistance vessels in hypertensive models (15, 16). Treatment of spontaneous hypertensive rats with arginase inhibitor (ABH) has been shown to decrease blood pressure and improve vascular function (2, 13, 14).

Both ARG1 and ARG2 are expressed constitutively in vascular tissues (7, 9). However, ARG1 has been shown to modulate vascular tone in disease conditions such as diabetes (10, 11), ischemia reperfusion (17), and hypertension (3, 13). However, the relative contribution of the ARG isoforms in salt-sensitive hypertension remains to be determined. To address this issue, we used a genetic mouse model with either partial deletion of the ARG1 gene or complete deletion of ARG2 gene. We could not examine complete ARG1 knockout (KO) mice as they do not survive beyond 2 weeks of age due to disruption of the hepatic urea cycle and hyperammonemia (18). We hypothesized that ARG1 isoform is involved in increased blood pressure, reduced vasodilator activity, and increased reactivity to constrictor stimuli in deoxycorticosterone acetate (DOCA)-salt hypertensive mice, contributing to mineralocorticoid hypertension.

## Materials and Methods

### Animals

All procedures were conducted in concordance with the guiding principles in the care and use of animals, approved by the Georgia Regents University Committee on the use of Animals in Research and Education. Mice lacking one copy of ARG1^+/−^ or both copies of ARG2^−/−^ in a C57BL/6J background at 12 weeks of age were used in this study. The animals were housed on a 12-h light/dark cycle and fed a standard chow diet with water or saline *ad libitum*. An expanded Section “Materials and Methods” is available in the online data supplement.

### DOCA-salt hypertension

Partial ARG1^+/−^, or complete ARG2^−/−^ KO or control wild type (WT) mice were unilaterally nephrectomized, and DOCA (200 mg/mouse) pellets were implanted SC in the scapular region. DOCA mice received water containing 1.0% NaCl and 0.2% KCl for 6 weeks. Control mice were unilaterally nephrectomized and received silastic pellets without DOCA and tap water.

### Systolic blood pressure measurements

Systolic blood pressure (SBP) was measured by tail cuff plethysmography (RTBP1001 system, Kent Scientific Corporation, Conn.) in conscious mice before and under DOCA treatment once per week thru the 6 weeks of treatment. At the end of the treatment, mice were euthanized, and aorta and mesenteric artery (MA) was isolated for further studies (see below).

### Vascular functional studies

After euthanasia, thoracic aortas, and second-order branches of MA were removed and cleaned from fat tissue in ice-cold physiological saline solution. Arterial segments of aorta and MA were carefully mounted as ring preparations in myograph chambers (Danish Myo Technology A/S) filled with physiological saline solution at 37°C (pH 7.4) and continuously bubbled with 5% CO_2_ and 95% O_2_. Isometric force was recorded using a powerLab/8SP data system (AD Instruments, Colorado Springs, CO, USA). Tissues were adjusted to maintain a passive force of 5 mN for the aortic and 3 mN for the second-order MA rings. Vessels were equilibrated for 60 min before experiments.

After equilibration, arterial segments were contracted with KCl (80 mM) to verify viability of preparations. After washing out KCl, endothelium integrity was assessed by contracting the segments with phenylephrine (PE, 1 μM; α_1_-adrenergic receptor agonist), followed by stimulation with acetylcholine (ACh; 1 μM; an endothelium-dependent vasodilator). Concentration-response curves to ACh (0.001–10 μM) were obtained in aorta or MA after precontraction with PE (1 μM). Then, following construction of control concentration-response curves to ACh in unilaterally nephrectomized or DOCA WT mice, tissues were washed several times, incubated with an ABH (100 μM, 60 min), and then a second curve was generated. Cumulative concentration-response curve to sodium nitroprusside (SNP, 0.0001–3 μM; a NO donor) were also performed in aorta and MA precontracted with PE. Additionally, concentration-response curves to PE (0.001–100 μM) were also performed in aorta or MA.

### Vascular arginase activity assay

Aorta and MA were collected and frozen in liquid nitrogen. Tissues were pulverized, homogenized in ice-cold lysis buffer (combined 1:4 w/v with 50 mM, Tris-HCl, 100 μM, EDTA, and EGTA, pH 7.5) containing protease inhibitor, PMSF, phosphatase inhibitors cocktail 2 and 3. Homogenates were sonicated and centrifuged at 14,000 × *g* for 20 min at 4°C and supernatants were collected for enzyme assay. Twenty-five microliters of supernatants in triplicate were added to 25 μL of Tris-HCl 121 (50 mM, pH 7.5) containing MnCl_2_ (10 mM) and the mixture were activated by heating for 10 min at 55–60°C. ARG activity was assayed by measuring urea production from l-arginine as previously described (19).

Additionally, aortas from WT Sham or DOCA mice were treated with ABH (100 μM) for 60 min, then collected and frozen for ARG activity assay.

### Western blot analysis

Protein (20 μg) extracted from aortas were separated by electrophoresis on a 10% SDS-polyacrylamide pre-cast gel and transferred to polyvinylidene difluoride membrane. Non-specific binding sites were blocked with 5% bovine serum albumin (BSA) in Tris-buffered saline/Tween for 1 h at 24°C. Membranes were incubated with primary antibodies (anti-ARG1, BD Transduction Laboratories, 1:1000; anti-ARG2, Santa Cruz Biotechnology, Inc., 1:250, Cell Signaling Technology, Inc.) overnight at 4°C. After incubation with secondary antibodies, signals were visualized using an enhanced chemiluminescence kit (Amersham, Piscataway, NJ, USA), and quantified by densitometry. Results are normalized to total actin protein and expressed as arbitrary unit.

### Coronary perivascular fibrosis

Hearts were embedded in paraffin blocks after fixation in 10% formalin. Paraffin-embedded sections (5 μm thick) were deparaffinized with xylene and rehydrated by immersion in a graded series of ethanol washes. Sections were stained by Picrosirius red following manufacturer’s protocol (Accustain Kit, Sigma-Aldrich). Collagen deposition around the coronary vessels was detected by red staining. The area of collagen staining relative to the vessel surface area was quantified using ImageJ (NIH). Perivascular fibrosis data are expressed as the collagen-to-vessel surface area ratio.

### Drugs and solutions

Physiological saline solution of the following composition was used: (in mM: NaCl, 118; NaHCO_3_, 25; glucose, 5.6; KCl, 4.7; KH_2_PO_4_, 1.2; MgSO_4_⋅7 H_2_O, 1.17; and CaCl_2_⋅2 H_2_O, 2.5). ACh, SNP, PE, phosphatase inhibitor cocktail 1 and 2 and protease inhibitor were purchased from Sigma-Aldrich (St. Louis, MO, USA). The ABH was obtained as a gift from Dr. Daniel Berkowitz. All of the reagents were of analytic grade. Stock solutions were prepared in deionized water.

### Data analysis

Results are presented as mean ± SEM, and n represents the number of animals used in the experiments. Relaxation or contraction values were calculated relative to the maximal changes from the contraction produced by PE and KCl, respectively, taken as 100% in each tissue. Concentration-response curves were fitted using a non-linear interactive fitting program (Graph Pad Prism 4.0; GraphPad Software Inc., San Diego, CA, USA), and two pharmacological parameters were obtained: the maximal effect generated by the agonist (or *E*_max_) and the negative logarithm of the concentration of agonist that produces 50% of the maximum response [−log EC_50_ (or pEC_50_)]. ARG activity data is represented as percent change respective to the control (100%). Student’s *t*-test or one-way analysis of variance (ANOVA) followed by Bonferroni *post hoc* test was used to evaluate the results. *P* < 0.05 was considered significant.

## Results

### Body and heart weight

**Figure 1:**
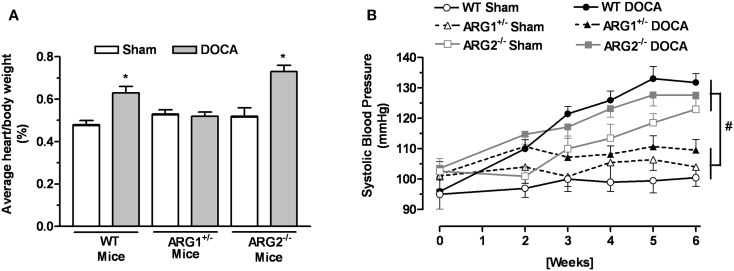
**Profile of arginase knockout (KO) mice in DOCA-salt treatment**. Ratio between heart and body weight **(A)** and systolic blood pressure (SBP) **(B)** of WT uninephrectomized (WT Sham), WT uninephrectomized and DOCA-salt treated (WT DOCA), partial arginase 1 (ARG1^+/−^) KO uninephrectomized (ARG1^+/−^ Sham), ARG1^+/−^ KO and DOCA-salt treated (ARG1^+/−^ DOCA), arginase 2 (ARG2^−/−^) uninephrectomized (ARG2^−/−^ Sham), and ARG2^−/−^ DOCA-salt treated mice. *P < 0.05, compared with its control Sham mice. ^#^*P* < 0.05, comparing upper and lower sets of SBP levels.

The body weight of WT and ARG genotype control mice and treatment groups ranged from about 25–27 g and did not differ among them (Table 1). The heart weight/body weight ratio was elevated in the DOCA-salt treatment groups of WT and ARG2^−/−^ mice vs. their respective Sham controls (Figure 1A). However, this ratio was not different between DOCA and ARG1^+/−^ Sham mice.

**Table 1:** **Body weight (g) did not differ among WT, ARG1^+/^^−^, and ARG2^−/^^−^ mice in uninephrectomized (WT Sham) or DOCA-salt treated mice**.

	WT Sham	WT DOCA	ARG1^+/−^Sham	ARG1^+/−^DOCA	ARG2^−/−^Sham	ARG2^−/−^DOCA
Body weight (g)	27 ± 2	27 ± 1	26 ± 3	25 ± 2	27 ± 2	26 ± 2
Aorta *E*_max_ (%)	98 ± 1	101 ± 3	97 ± 1	93 ± 2	96 ± 2	95 ± 3
Aorta pEC_50_	8.26 ± 0.07	8.37 ± 0.09	8.14 ± 0.09	8.09 ± 0.07	8.10 ± 0.07	8.17 ± 0.06
MA *E*_max_ (%)	100 ± 2	103 ± 2	101 ± 2	99 ± 1	101 ± 1	98 ± 2
MA pEC_50_	8.40 ± 0.05	8.51 ± 0.06	8.33 ± 0.08	8.26 ± 0.09	8.43 ± 0.05	8.37 ± 0.09

*The maximal effect (*E*_max_) and potency (pEC_50_) values obtained from concentration-response curves to a nitric oxide donor, sodium nitroprusside (SNP, 0.0001–3 μM) in aorta and second-order branches of mesenteric arteries (MA) from WT, ARG1^+/−^, and ARG2^−/−^ mice in uninephrectomized (WT Sham) or DOCA-salt treated mice. Data represent the means ± SEM of four experiments*.

### Systolic blood pressure

No differences in SBP measurements were observed among control WT and arginase KO mice before beginning the experiment at 12 weeks of age; SBP values ranged from 95 ± 5 to 104 ± 4 mmHg. After 2 weeks of DOCA treatment, SBP values for WT, ARG1^+/−^, and ARG2^−/−^ mice were higher than their Sham control (Figure 1B). During the third through the sixth week of DOCA-salt treatment, SBP values in WT and ARG2^−/−^ mice were steadily increased, whereas SBP in ARG1^+/−^ mice fell back toward control levels over this period (109 ± 4 vs. 101 ± 3 mmHg, respectively). Of particular note, SBP values for ARG2^−/−^ Sham mice rose progressively and slightly less than those for ARG2^−/−^ DOCA mice over the 6-week period, reaching values close to those for WT-DOCA mice. A progressive rise of SBP in non-treated ARG2^−/−^ mice has been reported (20).

### Vascular arginase activity and expression

**Figure 2:**
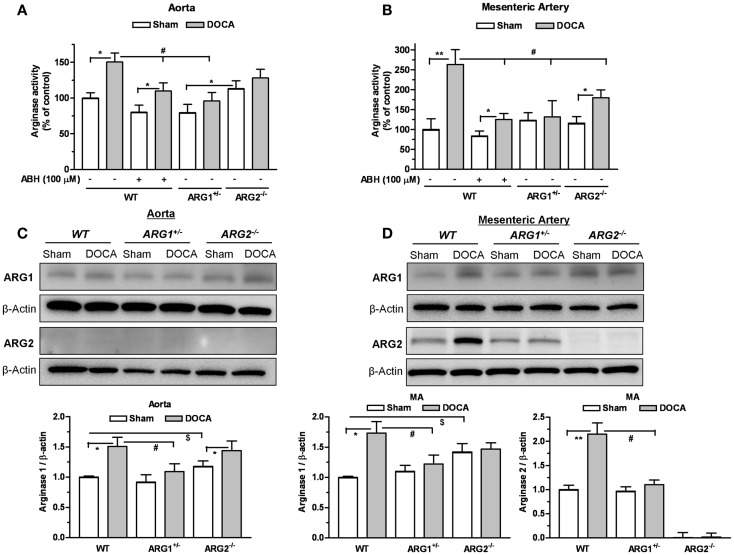
**Partial deletion of arginase 1 (ARG1^+/−^) or inhibition of arginase prevents DOCA-salt-induced increase in vascular arginase activity/expression**. Arginase activity measured in aorta **(A)** and mesenteric artery (MA) **(B)** in WT uninephrectomized (WT Sham), WT uninephrectomized and DOCA-salt treated (WT DOCA), partial arginase 1 (ARG1^+/−^) knockout uninephrectomized (ARG1^+/−^ Sham), ARG1^+/−^ and DOCA-salt treated (ARG1^+/−^ DOCA), arginase 2 (ARG2^−/−^) uninephrectomized (ARG2^−/−^ KO Sham), and ARG2^−/−^ KO DOCA-salt treated mice. Pretreatment with an inhibitor of arginase (ABH, 100 μM) prevented elevation of arginase activity in aorta and MA in WT-DOCA-salt treated mice **(A,B)**. Measurement of protein expression of ARG1 and ARG2 in aorta **(C)** and MA **(D)** of animals treated with Sham or DOCA-salt treated WT, ARG1^+/−^, or ARG2^−/−^ mice. Arginase activity in WT Sham group was considered as 100%. Data represents mean ± SEM of five to seven experiments. *P < 0.05, **P < 0.01, compared with its respective Sham group. ^#^*P* < 0.05, compared with WT-DOCA group. ^$^*P* < 0.05, compared with WT Sham group.

Arginase activities were elevated in DOCA WT aorta (1.5-fold) and MA (2.6-fold) compared to those of Sham WT mice (Figures 2A,B). Treatment with ABH (100 μM) reduced these elevations in vessels from WT mice. In ARG1^+/−^ mice, DOCA treatment did not elevate ARG activity in aorta (Figure 2A) or MA (Figure 2B) vs. those of Sham ARG1^+/−^ mice. In ARG2^−/−^ DOCA mice, ARG activity was elevated in MA (by 1.55-fold), but not in aorta, compared to ARG2^−/−^ Sham tissue (Figures 2A,B). Elevation of ARG activity induced by DOCA treatment in WT vessels was greater than observed in ARG1^+/−^ aorta and ARG2^−/−^ aorta and MA, but was not different from that in ARG 2^−/−^ DOCA aorta.

Parallel experiments assessing protein levels of ARG1 showed significant increases in the DOCA WT aorta by 1.49-fold (Figure 2C) and in the MA by 1.73-fold (Figure 2D) compared to WT Sham. Protein levels of ARG1 were not increased in aorta or MA from DOCA ARG1^+/−^ mice, but were elevated in aorta of DOCA ARG2^−/−^ mice compared to their Sham tissue (Figures 2C,D). In DOCA WT mice, ARG2 protein was markedly increased in MA (by 2.15-fold) (Figure 2D). ARG 2 expression was not detected in WT aorta. Basal ARG1 expression in vessels from ARG2^−/−^ mice was elevated above that of WT vessels.

Treatment of WT-DOCA aorta with ABH did not prevent elevation of ARG1 protein (not shown). These results indicate that ARG1 protein levels and activity are elevated by DOCA treatment.

### Vasocontractile responses to phenylephrine

**Figure 3:**
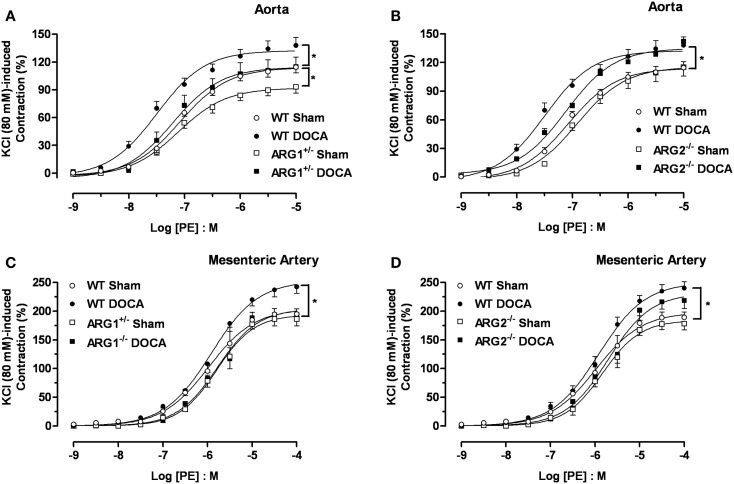
**Differences in contractile responses induced by phenylephrine (PE) in DOCA-salt treated arginase knockout (KO) mice**. Concentration-response curves to PE (0.001–10 μM) in WT uninephrectomized (WT Sham), WT uninephrectomized and DOCA-salt treated (WT DOCA), partial arginase 1 (ARG1^+/−^) KO uninephrectomized (ARG1^+/−^ Sham), ARG1^+/−^ and DOCA-salt treated (ARG1^+/−^ DOCA), arginase 2 (ARG2^−/−^) uninephrectomized (ARG2^−/−^ Sham) and ARG2^−/−^ DOCA-salt treated mice in aorta **(A,B)**, and mesenteric artery [MA; **(C,D)**]. Experimental values were calculated relative to the maximal changes from the contraction produced by KCl (80 mM), which was taken as 100%. Data represents mean ± SEM of five experiments. *P < 0.05, compared with WT Sham group.

Concentration-response curves to PE were performed to determine ARG genotype-related regulation of vascular reactivity to contractile stimuli. Aortas from WT-DOCA-salt mice displayed a greater vasoconstriction to PE compared with WT Sham tissues [maximum efficacy (*E*_max_) of 138 ± 8 and 114 ± 6%, respectively]. Contractile responses to PE were lower in the ARG1^+/−^ Sham (*E*_max_: 93 ± 6%) compared with those of WT Sham group, but were elevated in ARG1^+/−^ DOCA mice to the level of WT Sham mice (Figure 3A). Aortas from ARG2^−/−^ mice exhibited similar PE-induced contraction to those exhibited by aortas from WT Sham and DOCA-salt mice (Figure 3B).

As in aorta, the maximal contractile response to PE was higher in MA from WT DOCA than in WT Sham MA (*E*_max_: 243 ± 12 and 195 ± 9%, respectively). However, no differences in PE-induced contraction were observed in MA between WT Sham and ARG1^+/−^ Sham or DOCA mice (Figure 3C). Contractions induced by PE in MA were similar between WT Sham and ARG2^−/−^ Sham and between WT-DOCA and ARG2^−/−^ DOCA groups (Figure 3D).

### Vasorelaxation responses to acetylcholine and sodium nitroprusside

**Figure 4:**
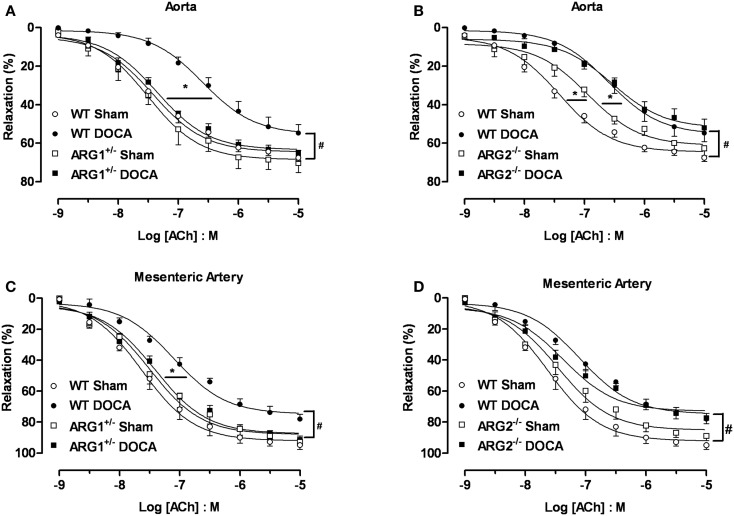
**Partial deletion of arginase 1 (ARG1^+/−^) prevents DOCA-salt-induced endothelial dysfunction in aorta and mesenteric artery (MA)**. Concentration-response curves to acetylcholine (ACh, 0.001–10 μM) in aorta **(A,B)** and MA **(C,D)** from WT, ARG1^+/−^, or ARG2^−/−^ mice in uninephrectomized (WT Sham) or DOCA-salt treated mice. Data were calculated relative to the maximal changes from the contraction produced by phenylephrine (PE, 1 μM), which was taken as 100%. Data are means ± SEM of six to eight experiments. *P < 0.05, indicates differences in pEC_50_ values of the dose-response curves. ^#^*P* < 0.05, compared with WT Sham group.

Endothelial dysfunction is a well-established feature of the DOCA-salt hypertensive animal model (21, 22). To determine the effect of ARG genotype on vascular function, we compared vasorelaxation responses to ACh (0.001–10 μM) in aorta from WT, ARG1^+/−^, or ARG2^−/−^ mice after 6 weeks of DOCA-salt with those in Sham mice. Similar aortic vasorelaxation responses to ACh were observed between WT and ARG1^+/−^ Sham mice (*E*_max_: 68 ± 2%; pEC_50_: 7.42 ± 0.09 and *E*_max_: 70 ± 5%; pEC_50_: 7.50 ± 0.07 for WT and ARG1^+/−^ mice, respectively, Figure 4A). DOCA-salt markedly reduced maximal responses to ACh in WT aorta (*E*_max_: 55 ± 4%) compared to their Sham control (*E*_max_: 68 ± 2%), whereas vasorelaxation responses to ACh in ARG1^+/−^ Sham and DOCA-salt vessels were not different (*E*_max_: 70 ± 5 and 65 ± 2%, respectively) (Figure 4A). Aorta from ARG2^−/−^ Sham mice showed similar *E*_max_ values (63 ± 4%) but displayed impaired sensitivity (pEC_50_) to ACh compared with those of WT Sham mice (pEC_50_: 6.94 ± 0.09 and 7.42 ± 0.09 for vessels of ARG2^−/−^ and WT Sham, respectively). Aortas from ARG2^−/−^ DOCA mice exhibited an impaired ACh-induced maximum vasorelaxation vs. their sham control (pEC_50_: 6.54 ± 0.07; *E*_max_: 52 ± 5%) which was not different from that of WT-DOCA aorta (Figure 4B).

The MA of WT-DOCA mice displayed impaired vasorelaxation to ACh (*E*_max_: 78 ± 3%) compared to WT Sham (*E*_max_: 95 ± 3%), but this impairment was absent in MA of ARG1^+/−^ mice Sham and DOCA mice (*E*_max_ 93 ± 2 and 92 ± 2%, respectively) (Figure 4C). The MA from the ARG2^−/−^ DOCA mice exhibited impairment of endothelial cell (EC)-dependent vasorelaxation similar to that of MA from WT-DOCA mice (Figure 4D). As in aorta, there tends to be impaired relaxation to ACh in ARG2^−/−^ Sham MA.

**Figure 5:**
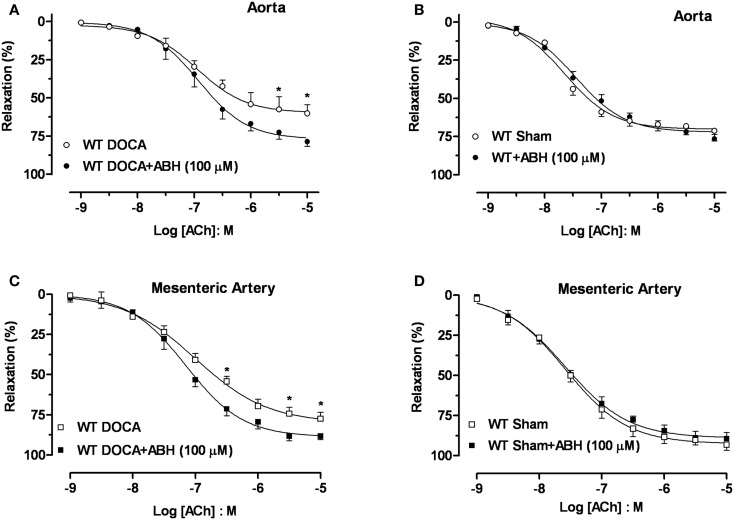
**Inhibition of arginase ameliorates DOCA-salt-induced endothelial dysfunction**. Treatment with an inhibitor of arginase (ABH, 100 μM; 60 min) prevented vascular endothelial dysfunction in DOCA-salt treated mice in aorta **(A)** and mesenteric artery [MA; **(C)**]. WT Sham vessels exposed to ABH showed no change in the ACh-induced vasorelaxation in aorta **(B)** and MA **(D)**. Data were calculated relative to the maximal changes in contraction produced by phenylephrine (PE, 1 μM), which was taken as 100%. Data are means ± SEM of four experiments. *P < 0.05, compared with WT-DOCA + ABH group.

Acute treatment with ABH (100 μM) significantly enhanced the *E*_max_ to ACh in aortas (Figure 5A) and MA (Figure 5C) from WT-DOCA mice (from 60 ± 5 to 79 ± 3% and from 76 ± 3 to 88 ± 2%, for aorta and MA, respectively). However, ABH did not alter the vasorelaxation to ACh in the WT Sham groups (Figures 5B,D).

Endothelium-independent relaxations in aorta or MA induced by the NO donor, SNP were not different between uninephrectomized (WT Sham) and DOCA-salt treatment in WT, ARG1^+/−^, and ARG2^−/−^ mice, respectively (Table 1).

### Coronary perivascular fibrosis

**Figure 6:**
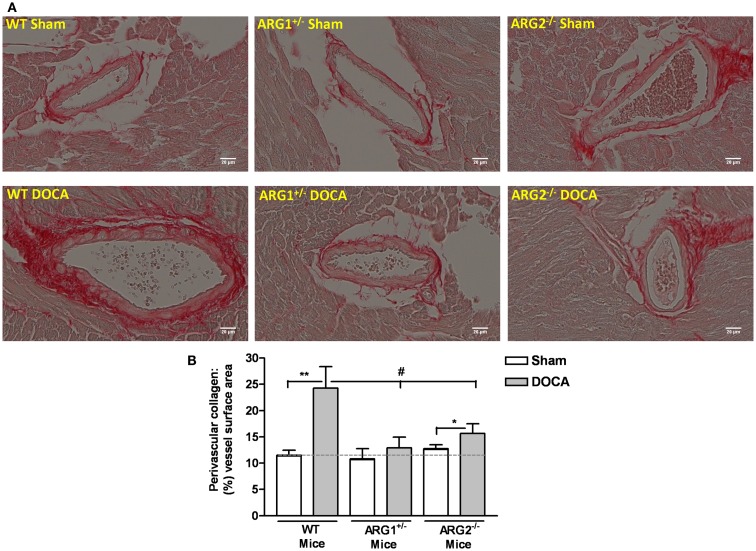
**Partial deletion of one copy of arginase 1 (ARG1) prevents coronary perivascular fibrosis in DOCA-salt treated mice**. Assessment of coronary perivascular fibrosis from WT, ARG1^+/−^, and ARG2^−/−^ mice in uninephrectomized (WT Sham) or DOCA-salt treated mice using picrosirius red staining (red color for collagen). **(A)** Shows a representative photograph of left ventricular paraffin-embedded tissue sections. Quantitative ratio of the perivascular collagen area to the vessel wall surface area is provided in **(B)**. Data are means ± SEM of three to four experiments. **P < 0.01, *P < 0.05, compared with its Sham group. ^#^*P* < 0.05, compared with WT-DOCA group.

Fibrosis was assessed as the amount of tissue collagen around the coronary arteries. WT-DOCA mice exhibited enhanced coronary perivascular fibrosis, as evident by the increased picrosirius red stain of collagen around the coronary vessel compared with control WT mice (Figure 6A). Importantly, collagen staining was not increased in ARG1^+/−^ DOCA mice compared to Sham control. Additionally, the ratio of coronary perivascular fibrosis to total vessel surface area increased markedly for WT-DOCA (2.1-fold) and significantly, but to a lesser extent (1.22-fold) in the ARG2^−/−^ DOCA vs. Sham mice, while no significant alteration in this ratio was observed in DOCA ARG1^+/−^ or Sham mice (Figure 6B). Our data indicate that increase ARG activity is associated with increased collagen production.

## Discussion

The major findings of this study are the contributions of arginase (ARG1 and ARG2) to elevated blood pressure, impaired EC-dependent vasorelaxation, increased vasoreactivity to constrictor stimuli, and enhancement of coronary perivascular fibrosis in a model of DOCA-salt hypertension. Several important observations have been made in our study. First, we show that SBP levels in ARG1^+/−^ DOCA do not rise as they do in WT-DOCA mice. The rise in SBP in WT-DOCA mice is similar to that reported by others (23, 24). In contrast, both ARG2^−/−^ Sham and DOCA mice exhibited progressive elevation in SBP, with a slightly greater rise in DOCA mice. A progressive hypertension has been reported for ARG2 KO mice (20). Second, vascular ARG activity is increased in aorta and second-order resistance MA from WT-DOCA-salt mice. Reduction or lack of the ARG1 or ARG2 genes prevented the DOCA-induced increase in aortic ARG activity, but only in ARG1^+/−^ aorta was activity lower than in WT aorta. Third, in WT-DOCA mice, protein levels of ARG1 are increased in aorta and both ARG1 and ARG2 are up-regulated in MA. Fourth, DOCA-induced impairment of endothelium-dependent vasorelaxation in aorta and MA is prevented in ARG1^+/−^ DOCA mice, and partially so in ARG2^−/−^ compared with its Sham control. These findings indicate that both ARG isoforms contribute to DOCA-induced vascular dysfunction, to varying degrees. Fifth, augmented contractile responses to the α-1-adrenergic agonist PE occur in aorta and MA from DOCA treated WT, ARG1^+/−^, and ARG2^−/−^ mice. Sixth, coronary perivascular fibrosis in DOCA-salt mice is prevented or reduced by deletion of ARG1^+/−^ or ARG2^−/−^, respectively.

Our findings indicate that enhancement of vascular ARG activity induced by ARG1 isoform has a key role in salt-induced hypertension. Salt sensitivity is associated with almost half of the cases of human hypertension (25). DOCA-salt hypertension is volume-dependent and is accompanied by low plasma renin-angiotensin system activity. The combination of DOCA-salt and unilateral nephrectomy result in hypertension and cardiac and renal hypertrophy. Increased vascular ARG has been linked in animal model for hypertension (3, 15, 16). The two isoforms of ARG have been shown to have different intracellular and tissue distributions (4, 7, 26). Depending on the disease state and tissue, ARG1, ARG2, or both may be elevated and exert prominent actions. Earlier studies have demonstrated up-regulation of both ARG isoforms in gracilis muscle arterioles and in aorta from Dahl-salt-sensitive (16) and spontaneously hypertensive rat (27). However, only ARG1 is reported to be increased in aorta from DOCA-salt rats (15), coronary arteries from pigs with aortic coarctation (13) and MA from genetic hypertensive rats (2). Our data show that absence of one copy of ARG1 gene prevents rises in SBP, indicating its involvement in DOCA hypertension. The rise in blood pressure in ARG2^−/−^ mice complicates assessment of ARG2’s role in DOCA hypertension. Absence of both ARG2 gene copies partially reduced but did not prevent elevation of SBP in DOCA-salt mice.

Phenylephrine-induced contractions in aorta from ARG1^+/−^, but not ARG2^−/−^, Sham mice were less than WT Sham mice, suggesting that activity of ARG1, but not ARG2, decreases aortic endothelial NO release in response to PE. A reduced contractile response was not observed in MA of ARG1^+/−^ Sham mice, possibly due to activity of ARG2. Importantly, contractile responses in aorta of all the mouse genotypes were enhanced by DOCA treatment, indicating that the DOCA-induced enhanced contraction did not involve either ARG isoform.

Deoxycorticosterone acetate-induced vascular endothelial dysfunction was largely absent in aorta and MA of ARG1^+/−^ mice. DOCA-induced dysfunction in ARG2^−/−^ was less pronounced vs. its sham controls, as the ARG2^−/−^ sham displayed a degree of impairment vs. WT sham. Thus, this isoform may contribute to vascular endothelial dysfunction. An important question is, how does lack of ARG2 cause this dysfunction.

Arginase can compete with NOS for the common substrate l-arginine in the vasculature. Increased vascular ARG activity/expression and decreased NO production have been observed in hypertension and diabetes (3, 10, 13, 19). The products of ARG action on l-arginine are urea and ornithine (28). Elevated ornithine levels from excessive ARG activity also could contribute to pathological vascular fibrosis and thickening by increasing the formation of polyamines and proline from ornithine. Polyamines and proline promote cell growth and collagen formation, respectively (29, 30). Our findings demonstrate that DOCA-salt induces cardiac hypertrophy and increases perivascular collagen deposition in WT mice, and that these effects are absent in ARG1^+/−^ mice. Lack of ARG2 also reduces DOCA-induced perivascular fibrosis. Long term treatment of SHR with an ABH has been shown to reduce blood pressure and cardiac fibrosis (2). Our data suggest that increased ARG1 mediates cardiac hypertrophy and perivascular fibrosis through increased synthesis of polyamines and proline. Elevated blood pressure/vascular resistance also contribute to cardiac hypertrophy. Earlier studies have demonstrated that hypertension, diabetes, atherosclerosis, and aging are associated with elevated vascular stiffness/decreased arterial compliance, which is recognized as an important and independent cardiovascular risk factor (31). Further studies are needed to determine whether reduction of ARG1 gene expression diminishes vascular stiffness via decreased levels of ornithine, polyamines, and proline.

The pathogenesis of DOCA-salt hypertension in conduit arteries is reported to involve increased reactive oxygen species (ROS) production (32). Superoxide and peroxynitrite contribute to increased vascular ARG activity, decreased l-arginine availability to eNOS and its uncoupling, reduced NO production, and even greater levels of these ROS (33, 34). Both peroxynitrite and hydrogen peroxide increase endothelial ARG expression/activity and inhibition of NADPH oxidase blocks this action of hydrogen peroxide (34). NOS uncoupling also would limit the production of *N*^G^-hydroxyl-l-arginine (NOHA), an intermediate product in the generation of NO and a potent inhibitor of ARG. Inhibition of NADPH oxidase by apocynin has been reported to prevent elevation of ARG activity and largely prevent a lipopolysaccharide (LPS)-induced increase in ARG1 mRNA in rat alveolar macrophages (35). Additionally, exposure of cultured rat retinal cells to LPS also strongly induces expression of ARG1, which is markedly reduced by apocynin co-treatment (36). It is probable that ROS are involved in the elevation of ARG1 activity and expression observed in our study.

In our model, DOCA treatment progressively increased SBP in WT and ARG2^−/−^ mice. Moreover, SBP also rose in the Sham ARG2^−/−^ mice verses Sham WT mice. Huynh et al. (20) have previously reported a progressive rise in blood pressure in ARG2^−/−^ KO mice along with increased plasma levels of the norepinephrine (NE) precursor (dihydroxyphenylalanine) and its metabolite (dihydroxyphenylglycol). Their data suggest that sympathetic nervous activity is increased in the cardiovascular system and responsible for elevated blood pressure in these ARG2^−/−^ mice. Further study is needed to more clearly define how ARG2 regulates blood pressure.

In summary, our results showed that ARG is involved in the pathogenesis of hypertension. Using a genetic approach, we conclude that both ARG isoforms contribute to vascular endothelial dysfunction in DOCA-salt hypertension.

## Conflict of Interest Statement

The authors declare that the research was conducted in the absence of any commercial or financial relationships that could be construed as a potential conflict of interest.
